# Chromatin-Associated Pea Apyrase psNTP9 Function as a DNA-Binding Regulatory Protein in Yeast and Arabidopsis

**DOI:** 10.3390/plants14223514

**Published:** 2025-11-18

**Authors:** Huan Wang, Robert D. Slocum, Xingbo Cai, Greg Clark, Stanley J. Roux

**Affiliations:** 1 Department of Molecular Biosciences, The University of Texas at Austin, Austin, TX 78712, USA; huanwang2020@utexas.edu (H.W.); caixingbo2018@utexas.edu (X.C.); gbclark@utexas.edu (G.C.); sroux@austin.utexas.edu (S.J.R.); 2 Department of Biological Sciences, Goucher College, Towson, MD 21204, USA

**Keywords:** NTPDase, ChIP-seq, DNA-binding, Arabidopsis, apyrase, psNTP9-DM, *PHM6*

## Abstract

As reported in earlier work, when a pea apyrase, psNTP9 (PS), and a modified version of it, psNTP9-DM (DM), are expressed in *Saccharomyces cerevisiae*, they localize to nuclei, binding to largely non-overlapping promoter regions of chromatin. PS- and DM-expressing yeast also exhibit different expression profiles for potentially regulated target genes, consistent with observed phenotypes. In the present study, we use ChIP-seq assays to show that PS and DM also associate with largely different promoter regions of Arabidopsis genes, with similar non-overlapping expression profiles for potential target genes. Functional studies, using electrophoretic mobility shift assays (EMSA), verified PS-specific binding to yeast or plant promoter binding sites. DM binding to both heterologous dsDNA and to PS-specific binding site sequences was minimal. AlphaFold3 modeling of PS protein binding to a yeast *PHM6* promoter sequence identified potential DNA-binding residues and a potential binding site motif (5′-(G/T)GG(G/T)A-3′) that is also present in two Arabidopsis promoter binding sites. These novel findings extend the previously known functions of PS and other plant apyrases in the Golgi or extracellular matrix, and support their potential function as DNA-binding proteins that can regulate gene expression in both yeast and Arabidopsis.

## 1. Introduction

Apyrases (NTPDases; EC 3.6.1.5) hydrolyze NTP and NDP, removing their terminal phosphates. In animals, an important function of apyrases is the hydrolysis of extracellular ATP (eATP), thus modulating a wide range of physiological processes [1]. In plants, regulation of [eATP] by “ecto-apyrase” activities is also important in diverse growth and developmental processes [2,3,4]. In Arabidopsis, *AtAPY1* expression promotes auxin transport [5], enhances pollen tube growth [6], and regulates stomatal aperture [7] and abiotic stress responses [3]. Another important function of plant apyrases is maintaining the NDP/NMP balance in the Golgi, which is essential for efficient protein glycosylation [8], and apyrase activities in secretory vesicles may help regulate [eATP] in the extracellular matrix [4].

Plant apyrases have also been reported in nuclei [9,10], although their function in this organelle is largely unexplored. In etiolated seedlings, psNTP9 (“PS”), the pea ortholog of *AtAPY1*, was isolated from nuclei [9]. Ectopic expression of *psNTP9* resulted in an enhanced root system architecture, a drought-tolerance phenotype, and increased seed yields in both Arabidopsis and soybean [11,12]. Constitutive expression of *psNTP9* also increased phosphate contents of yeast, Arabidopsis seedlings, and “hairy root” cultures of corn, soybean, and canola [13,14]. Given the potential importance of these phenotypes for the development of drought-tolerant crops with improved P utilization efficiency, we have been further investigating the mechanisms by which PS regulates plant growth, with a focus on its nuclear functions.

Both PS and AtAPY1 are regulated by calmodulin (CaM) [15,16], and one of two potential CaM-binding sites on PS (PCBS2) was functionally characterized [15]. We introduced two mutations into the second potential CaM-binding site (PCBS1) in PS in an attempt to engineer enhanced CaM-binding to the enzyme, which is designated as the “double mutation” (“DM”) version of PS [14]. The PCBS1 sequence overlaps a predicted nuclear localization signal [17], suggesting that CaM binding to that site might regulate targeting of PS to the nucleus. Introduction of these mutations into PCBS1 might also influence nuclear targeting directly, absent CaM binding, or could alter the NTPDase activities of PS. Thus, the DM variant offered a tool to further investigate PS functions.

In a recent study, we generated transgenic lines ectopically expressing *PS* or *DM* in both yeast and Arabidopsis [14]. Purified DM from yeast did not exhibit increased CaM binding or significantly different NTPDase activities, compared with PS. Although PS and DM were both co-purified with nuclei and known chromatin-associated proteins, the amount of DM protein was lower than that of PS. Overexpression of either PS or DM produced significantly different gene expression profiles in both yeast and Arabidopsis, which helped explain the phenotypic effects of PS and DM on growth and phosphate uptake in both organisms. It also raised the question of whether PS and DM proteins have different chromatin binding sites. In yeast, ChIP-seq assays confirmed PS- and DM-specific binding in the promoters of many differentially expressed genes (DEG), suggesting that PS and DM might be functioning in transcriptional regulation. Enriched motifs for known transcription factors (TF) in these binding sites, and sets of potentially regulated target genes, were largely non-overlapping [14].

What remained unresolved was whether PS and DM also had distinct binding sites in Arabidopsis chromatin, and whether they differed in their ability to bind to specific promoter sequences in both yeast and Arabidopsis, either directly or indirectly, through interactions with other DNA-binding proteins. A favored approach to resolving the latter question is the electrophoretic mobility shift assay (EMSA). In this report, we used ChIP-seq assays to identify PS and DM binding sites in the promoters of Arabidopsis genes. Using EMSA, we functionally validated PS binding to promoter sequences for a yeast gene and two Arabidopsis genes that were differentially expressed in response to ectopic expression of *PS*. We further modeled potential DNA-binding sites using AlphaFold3 [18] to characterize possible interactions with enriched TF motifs in these sequences. The results support a potential role for PS and DM in transcriptional regulation of yeast and Arabidopsis genes and provide important new insights into apyrase functions.

## 2. Results

### 2.1. Targeting of PS to Plant Nuclei

**Figure 1:**
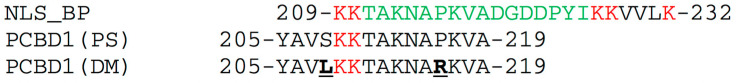
The bipartite nuclear localization signal (NLS_BP) overlaps with the putative calmodulin-binding domain 1 (PCBD1) in PS and DM proteins. The native PCBD1 sequence contains the S208L and P216R mutations in the DM protein (bold, underlined; [14]). The P216R mutation is located within the spacer region (green) of NLS_BP.

Hsieh et al. [17] cloned the nuclear-localized pea NTPase (psNTP9) and reported that residues 209–232, which overlap with PCBD1, were a potential bipartite nuclear localization signal (NLS_BP), as is shown in Figure 1. Although this sequence does not fit the consensus Prosite motif PS50079 of (R/K)-(R/K)-(X_10_)-(≥3 of 5 = R/K residues) for NLS_BP, Kosugi et al. [19] reported that spacer sequence lengths between 10 and 16 residues did not significantly change function in plant NLS_BP. Kosugi et al. [19] also noted that basic and hydrophobic residues are rare at the center of plant NLS-BP spacer sequences; thus, the P216R mutation introduced in the PCBD1-DM sequence and located in the center of the spacer sequence (green residues, Figure 1) may interfere with nuclear targeting. The approximately 2-fold lower amount of DM versus PS protein in purified Arabidopsis nuclei supports this possibility [14].

### 2.2. PS and DM Have Different Binding Affinities for Heterologous dsDNA

**Figure 2:**
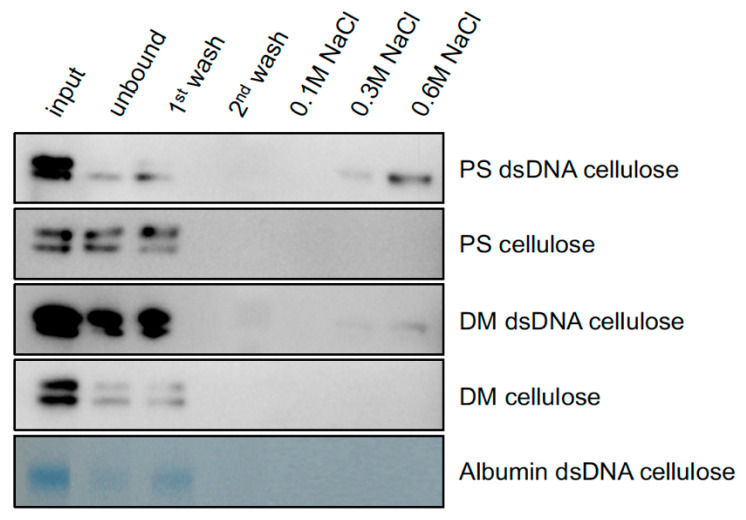
SDS-PAGE immunoblot analysis of the differential binding of PS and DM (4.8 µg loadings) to a dsDNA-cellulose column, and of their non-binding to a cellulose column as the negative control. PS and DM proteins were purified from yeast. Ovalbumin served as a negative protein control.

Purified preparations of yeast-expressed PS and DM were tested for their binding to a heterologous double-stranded DNA (dsDNA)-cellulose column and to a column of cellulose alone. PS and DM proteins were considered bound if they did not elute in the first wash (0.05 M NaCl). As judged by immunoblot analysis, at least 20% of the loaded PS bound to the dsDNA-cellulose (Figure 2). A small portion of the bound PS was eluted by 0.3 M NaCl, and significantly more of it was eluted by 0.6 M NaCl. Less than 2% of the loaded DM was bound to the dsDNA-cellulose. All of the bound DM was eluted by 0.6 M NaCl. This indicated that DM had a lower binding affinity to dsDNA cellulose. Neither PS nor DM protein bound to cellulose alone. Ovalbumin was used as a negative control and, as judged by Coomassie blue staining, more than 95% of the ovalbumin loaded onto the dsDNA column was either unbound or eluted in the low-salt (0.05 M NaCl) wash (Figure 2).

### 2.3. Identification of PS and DM Binding Sites in the Promoters of Arabidopsis Genes and Integration with Gene Expression Data

To investigate the genome-wide DNA-binding profiles of PS and DM proteins in *Arabidopsis thaliana*, we performed chromatin immunoprecipitation followed by high-throughput sequencing (ChIP-seq) using 7-day-old transgenic seedlings overexpressing 4Myc-tagged PS or DM proteins. High-confidence peaks were identified within 1–1500 bp upstream of transcription start sites (TSS) and filtered for genes that were differentially expressed (DE) in corresponding RNA-seq datasets for PS or DM seedlings grown under identical conditions.

Among all identified binding sites, 16,667 were PS-specific, 23,207 sites were DM-specific, and 28,889 represented PS and DM sites that overlapped to varying degrees. Of the total 39,874 PS- or DM-specific binding sites, 13,560 sites (34%) were located in promoters of 12,392 potentially regulated target genes (Table S1). Venn analysis of their potential target genes (Figure S1) showed that only 1625 (13.1%) of these genes exhibited both PS and DM sites in their promoters, while the vast majority of genes contained either PS- or DM-binding sites. 

Integration of ChIP-seq binding site and RNA-seq transcriptome datasets showed that approximately 4% of genes with PS- or DM-binding sites in their promoters were differentially expressed (DE) in PS- or DM-overexpressing seedlings, with a smaller number of binding sites being correlated with specific gene expression. For example, out of 213 genes with PS-binding sites in their promoters, only 140 (65.7%) were specifically expressed in PS seedlings, while 53 genes were DE only in DM seedlings, and 20 were DE in both PS and DM seedlings (Table S1). Similarly, only 67 out of 274 genes (24.4%%) with DM-specific binding sites in promoters were DE only in the DM seedlings, with the rest being DE in the PS seedlings or both PS and DM seedlings (Table S1).

### 2.4. Identification of Enriched Transcription Factor Binding Site Motifs in PS and DM Binding Sites of Arabidopsis Genes

We further characterized PS- and DM-binding sites using motif enrichment analysis performed using the MEME Suite v5.5.7 SEA tool against an Arabidopsis TF motif database. Among all high-confidence binding sites, 17 motifs in 10 different TF families were significantly enriched in PS-specific binding regions; 7 motifs in two TF families were enriched in DM-specific sites (Table 1). Dof TF motifs with an AAAG core sequence were found in 227 PS- and 34 DM-binding sites, making it the most abundant motif (Table 1). Motifs for the bZIP (ABI5) and MYB-related (MYBS1, SRM1) TF (Table 1), which regulate many genes involved in ABA synthesis and signaling [20,21], were statistically the most highly enriched motifs. Their over-representation only in DM-binding sites [14] suggests a potential role for DM in regulating ABA responses (Table S2).

Enriched motifs in PS binding sites are mostly for TF that regulate diverse developmental processes (Table 1). For example, LOB and JKD function in the specification of tissue and organ boundaries [22,23]. The enriched AATAATT motif for homeobox TF is found in 104 PS-binding sites in promoters of 41 unique genes. The promoter of TF HFR1, which regulates genes involved in phytochrome responses [24], has a perfectly conserved ATHB1/HAT5 motif, suggesting the possibility that PS binding might be involved in light-regulated development. GLABRA2 regulates epidermal cell identity [25] and ATHB53 regulates auxin/cytokinin signaling important for root development [26]. Interestingly, PS-binding sites in the promoters of 13 different genes were also enriched for SHN3 motifs. The SHN1-SHN3 TF clade activates genes involved in wax synthesis and cuticle formation in Arabidopsis [27]. A SHN3 motif is also found in the promoter of PER50, a secreted class III peroxidase that functions in H_2_O_2_ metabolism and lignification of the cell wall [28], processes which impact growth in Arabidopsis seedlings and are regulated, in part, by apyrase expression levels [29].

A list of PS-specific binding sites, enriched motifs, and expression profiles and annotation for potentially regulated target genes that were differentially expressed only in PS seedlings is provided in Table S3.

### 2.5. Functional Characterization of PS and DM Binding Site Motifs in Yeast and Arabidopsis Genes Using EMSA

We adopted the following scheme for selecting PS binding sites for functional studies. We considered only high-confidence binding sites in promoters of genes that were DE only in PS-overexpressing yeast or Arabidopsis seedlings. We then performed sequence enrichment analyses to identify potential TF motifs in those binding sites. Since individual promoters often exhibit multiple binding sites, each of which may contain multiple TF motifs, we selected the binding site with the highest-scoring motif sequence (highest similarity to the motif consensus sequence) for any given DE gene. Further selection criteria included binding site lengths >200 bp and TF motif locations approximately centered within the binding site sequence, which is normally observed for ChIP-seq peaks [30]. A final consideration was given to possible roles for potential PS-regulated target genes in PS-specific phenotypes observed in yeast and Arabidopsis seedlings that overexpress *PS*. This approach led to the selection of a binding site in the yeast *PHM6* gene, identified in a previous study [14]. We also selected two additional sites in Arabidopsis *WRKY42* and *PER50* promoters identified in the present study for *in vitro* DNA-binding studies based on EMSA [31]. PS-specific induction of these genes in RNA-seq assays was validated by qRT-PCR (Table S4).

#### 2.5.1. A PS Binding Site in the PHM6 Promoter Contains an E-Box Motif

Mapping of potential PS and DM binding sites in yeast shows that there is a PS binding site, but not a DM binding site, in the promoter region of *PHM6* (Figure S2). This binding site contains the CACGTG motif (E-box) for the yeast Pho4p transcription factor, which is a bHLH TF involved in regulating genes involved in phosphate metabolism in yeast (Austin and Mayer 2020; Yip et al. 2023) [32,33]. PHM6 (phosphate metabolism protein 6) is involved in phosphate transport and was recently suggested to function in concert with Pi transporters of the plasma membrane to increase Pi uptake in yeast under phosphate surplus conditions (Kulakovskaya et al. 2023) [34]. *PHM6* expression is induced in PS yeast but is not differentially expressed in DM yeast. This suggests that *PHM6* expression could be regulated by PS binding but not by DM binding to the promoter.

**Figure 3:**
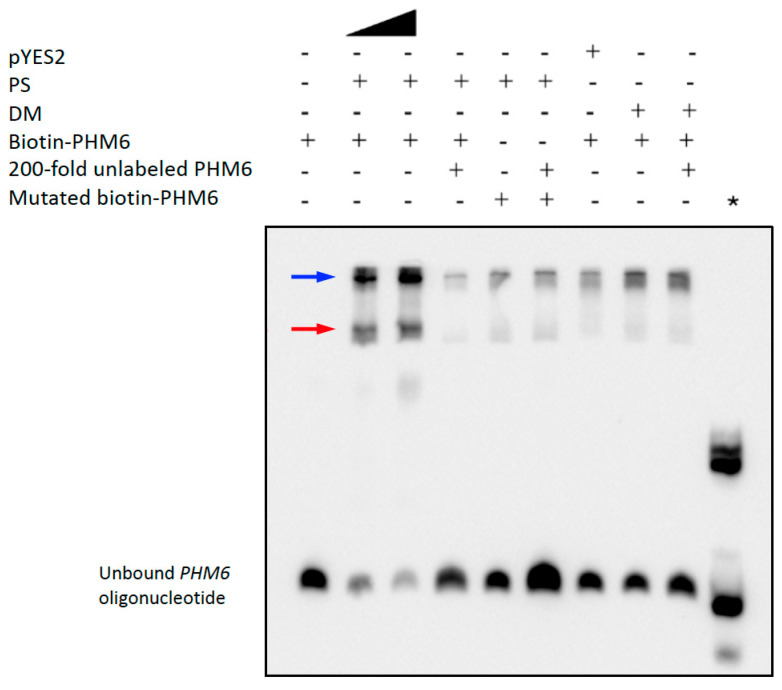
PS binds specifically to the yeast *PHM6* promoter. Sample constituents are indicated by (+). A total of 10 nmol of biotin-labeled probe was used in each lane, and a 200-fold excess amount of unlabeled *PHM6* duplex was used as the competitor. All assay tubes were incubated at room temperature for 20 min to allow for binding to occur. Protein extracted from the pYES2-expressing yeast was used as a negative control. Mutated *PHM6* oligo was also added to the reaction samples as a negative control. Samples were separated on a 6% polyacrylamide native gel. The black triangle represents a doubling of the amount of PS protein. The positive control (*) is Biotin-EBNA Control DNA with EBNA extract provided with the kit and used to validate the assay conditions. The red and blue arrows indicate reduced *PHM6* oligo mobilities resulting from different PS-specific binding interactions. Some low-mobility bands near the top of the gel represent non-specific *PHM6* binding by proteins in the pYES2 control, which are also present in the PS and DM samples. Their binding is not reduced by the excess competitor. The results shown in this figure are representative of two independent assays.

In order to validate yeast ChIP-seq data, we investigated whether PS could bind to a PS-specific binding site sequence in the *PHM6* promoter using a biotin-labeled PS binding site oligonucleotide EMSA. The assay identified two distinct bands with decreased mobilities in the PS sample (Figure 3, lane 2). Using twice as much PS protein (Figure 3, lane 3) produced a stronger binding signal and a weaker biotin-labeled *PHM6* signal. The addition of unlabeled *PHM6* competitor oligo decreased the amount of labeled oligo bound to PS (Figure 3, lane 4), supporting binding specificity. Mutated *PHM6* (no E-box motif), used as a negative control, did not produce these band shifts (Figure 3, lanes 5 and 6), and “pYES2 protein”, which was purified from the pYES2 empty vector strain by the same method used to purify PS and DM, did not produce the PS-dependent band shift (Figure 3, lane 7). The purified DM protein also did not exhibit significant binding to the *PHM6* oligo (Figure 3, lane 8). Since the purity of PS and DM proteins used in these and other EMSA was only ~1%, the two PS-specific bands (Figure 3, lanes 2 and 3) may represent DNA binding by PS complexed with other proteins. Other low-mobility bands present at the top of each lane may represent DNA-binding by contaminating yeast proteins, such as those seen in the pYES2 control sample.

#### 2.5.2. PS Binds to Arabidopsis WRKY42 and PER50 Promoter Sequences with Enriched TF Motifs

**Figure 4:**
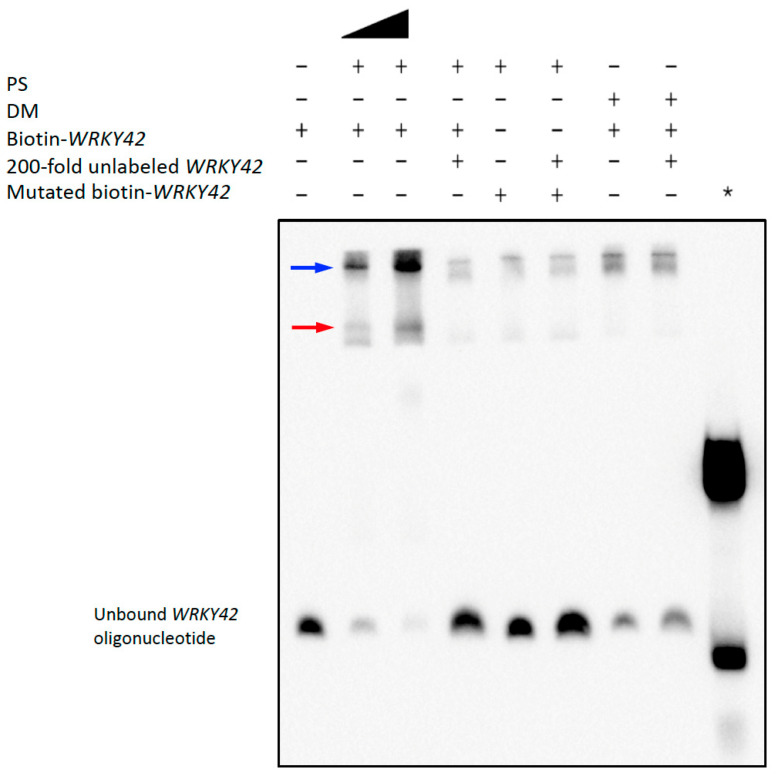
PS binds to the Arabidopsis *WRKY42* promoter. PS-dependent Arabidopsis *WRKY42* binding site shift was detected by EMSA. EMSA using biotin-labeled *WRKY42* DNA probes showed a PS concentration-dependent shift similar to yeast *PHM6* and Arabidopsis *PER50*, with no significant shift seen for DM. The black triangle represents a doubling of the amount of PS protein. The binding specificity was validated by competition with the excess unlabeled *WRKY42* probe and loss of binding with the mutated probe. The positive control (*) was Biotin-EBNA Control DNA with EBNA extract, which was used to validate the assay conditions. The red and blue arrows indicate reduced *WRKY42* oligo mobilities resulting from different PS-specific binding events. Some low-mobility bands near the top of the gel represent non-specific *WRKY42* binding, which is not decreased by the excess competitor. The results shown in this figure are representative of two independent assays.

**Figure 5:**
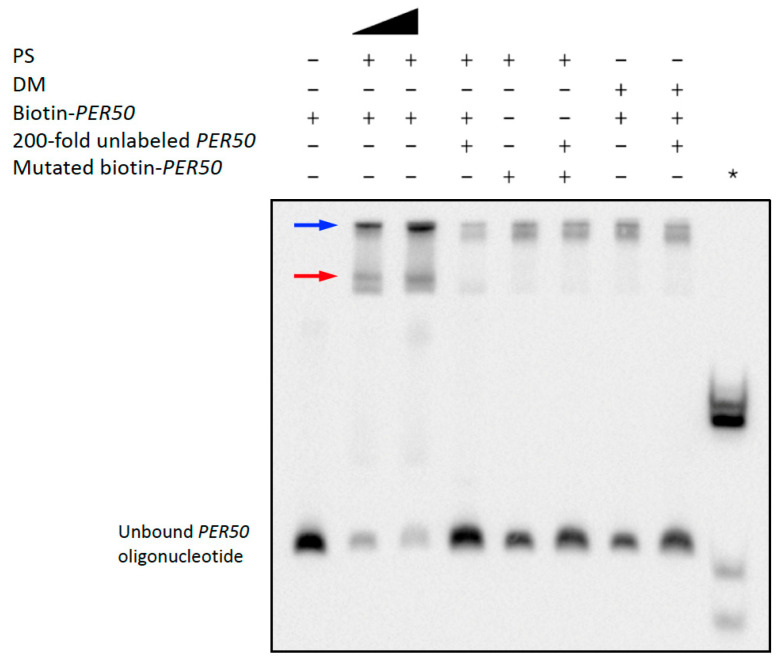
PS binds specifically to the Arabidopsis *PER50* promoter. The PS-dependent Arabidopsis *PER50* binding site shift was detected by EMSA. EMSA using biotin-labeled *PER50* DNA probes showed a PS concentration-dependent shift similar to yeast *PHM6* and Arabidopsis *WRKY42*, with no significant shift seen for DM. The black triangle represents a doubling of the amount of PS protein. The binding specificity was validated by competition with the excess unlabeled *PER50* probe and the loss of binding with the mutated probe. The positive control (*) was Biotin-EBNA Control DNA with EBNA extract, which was used to validate the assay conditions. The red and blue arrows indicate reduced *PER50* oligo mobilities resulting from different PS-specific binding events. Some low-mobility bands near the top of the gel represent non-specific *PER50* binding, which is not decreased by excess competitor. The results shown in this figure are representative of two independent assays.

To test whether PS binds *WRKY42* and *PER50* promoter sequences, we conducted EMSA using biotin-labeled PS binding-site sequences centered around enriched TF motifs. As shown in Figure 4, the PS sample produced clear, concentration-dependent band shifts with the *WRKY42* oligo containing a DOF1.5 motif (Figure S3), whereas DM did not. The addition of excess unlabeled *WRKY42* decreased the amount of labeled oligo bound by PS, demonstrating binding specificity, and no shift was observed when using a mutated *WRKY42* probe. Similarly, PS, but not DM, caused a distinct shift in the *PER50* probe containing a SHN3 motif (Figure 5 and Figure S4). Again, binding was competitively inhibited by the excess unlabeled probe and was abolished with a mutated *PER50* sequence. Additional band shifts likely resulted from the interactions with other DNA-binding proteins in the samples, as previously noted for the *PHM6* assays.

### 2.6. Co-Immunoprecipitation Analyses of PS- and DM-Interacting Proteins in Yeast and Arabidopsis

We performed co-IP assays and mass-spec identification of Myc-tagged PS- and DM-interacting proteins from crude yeast extracts prepared using a protocol similar to that used for the partial purification of PS and DM proteins. Parallel co-IP experiments were run with Arabidopsis seedling extracts. Annotated chromatin proteins or TF that co-IP with PS or DM proteins are listed in Table S5. In both yeast and Arabidopsis, two nucleolin-like peptidyl-prolyl *cis*-trans isomerases and a third nucleolin NUCL1 co-IP with both PS and DM. Nucleolins bind a variety of proteins and RNAs and are involved in ribosome biogenesis. It is unclear whether the interacting yeast DNA repair protein MSC1 binds DNA directly. Interestingly, out of 87 plant and 38 yeast interacting partners in co-IP samples, only a single transcription factor, Scarecrow-like protein 30 (SCL30), was identified in seedling samples. SCL30 is in the GRAS TF family, which interacts with a wide variety of *cis*-elements in genes that regulate diverse growth and stress responses [35].

### 2.7. Structural Basis for Differences in DNA-Binding by PS and DM Proteins

**Figure 6:**
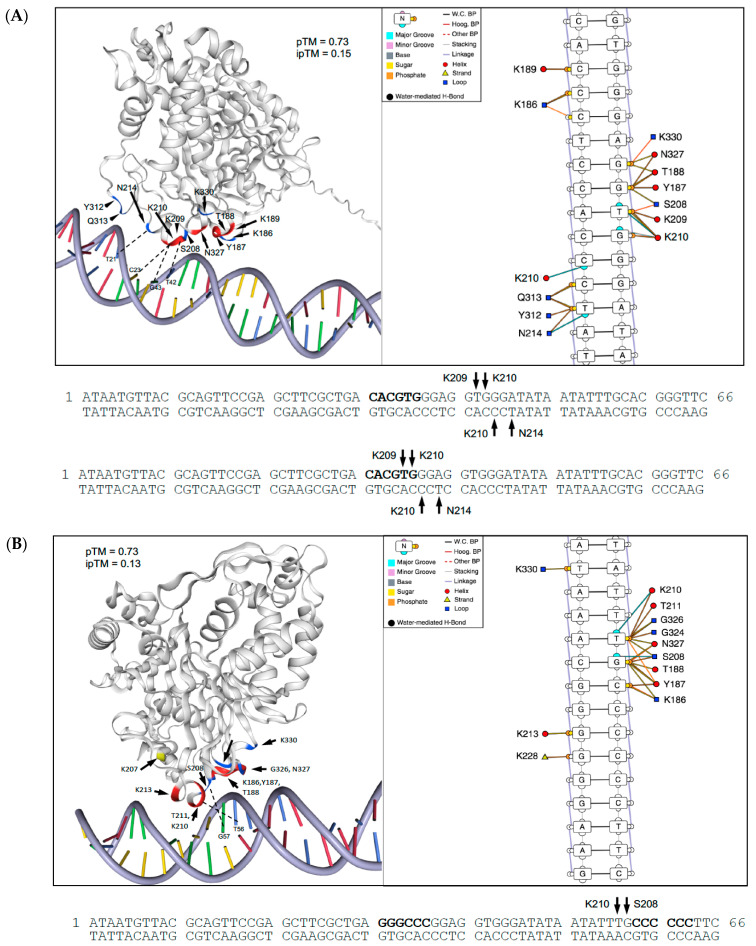

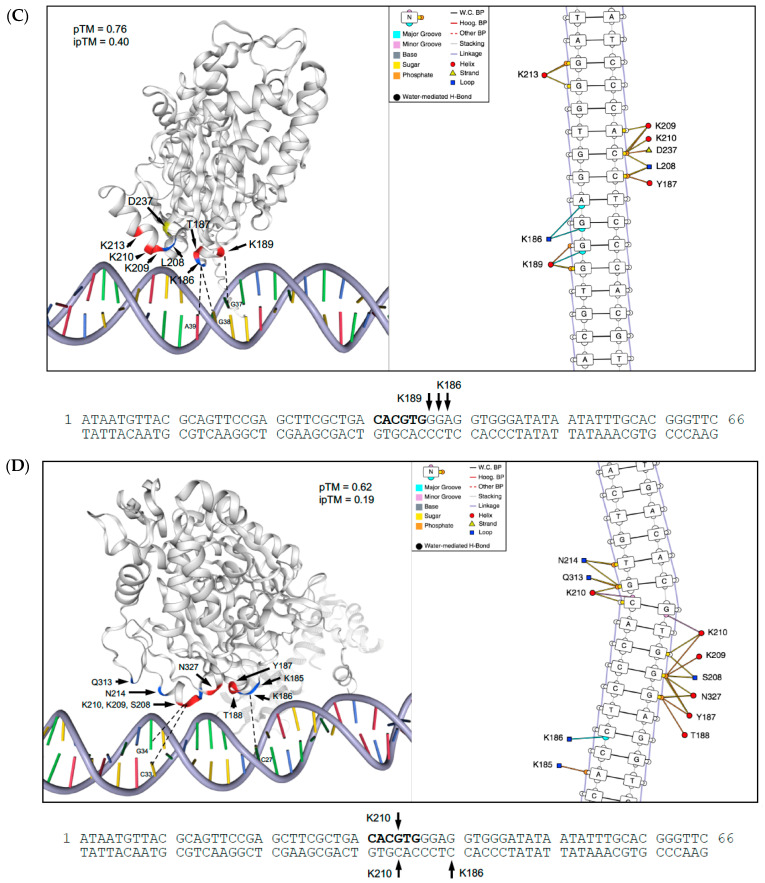
Modeling of mature PS and DM protein binding to *PHM6* oligonucleotide sequences using AlphaFold3 (AF3; [18]). Structures were then visualized, and binding site interaction maps were produced using DNAproDB (https://dnaprodb.usc.edu). For reference, sequence-specific binding is shown on the *PHM6* oligo sequence below the structure and interaction map. PS binding to the PHM6 test oligo is shown in (**A**). AF3 did not predict PS binding to an identical 5′-TGGGA-3′ sequence that overlaps with the CACGTG motif, as shown in the second oligo sequence map. Predicted PS binding to the mutated *PHM6* occurs near the 3′-end of the oligo (**B**). The S208L and P216R mutations in PCBD1 enhance its amphipathic α-helix character and alter DM binding to *PHM6*, decreasing interactions with nucleobases and the sugar-phosphate backbone adjacent to the CACGTG motif (**C**). In the presence of Ca^2+^, Arabidopsis Calmodulin 1 associates with the N-terminal sequence and PCBD2 α-helix of the PS protein. As in the DM model, the PCBD1 helix aligns with the minor groove, where the interaction with nucleobases C33 and G34 within the CACGTG motif was stabilized by backbone interactions in the major groove (**D**). The overall predicted TM (pTM) and interface-predicted TM (ipTM) confidence values for each model are indicated. pTM values between 0.62 and 0.76 are >0.5 cutoff typical for high-confidence protein structure predictions. ipTM values ranging from 0.13 to 0.40 are all <0.6 cutoff for high-confidence interface predictions.

We used AlphaFold3 [18] to model potential PS and DM binding to both test and mutant *PHM6* oligonucleotide sequences used in EMSA (Figure S6). Binding site interaction maps and supporting structure models are presented in Figure 6. These models support potential direct binding of PS to a ‘-TGGGA-3′ motif adjacent to (or overlapping with; see Figure 6 legend) the enriched CACGTG Pho4p motif in this promoter sequence (Figure 6A). PCBD1 residues K209, K210, and N214 facilitated sequence-specific interactions with major groove nucleobases. PS binding to the mutated oligo involved interactions between PCBD1 residues S208 and K210 and a 5′-TG-3′ sequence at the 3′-end of the *PHM6* oligo, stabilized by additional backbone interactions (Figure 6B). The altered PCBD1 structure seen in the DM protein (S208L, P216R mutations) resulted in its alignment with the minor groove and loss of nucleobase binding. DM residues K186 and K189 interacted with major groove nucleobases 5′-GGA-3′ adjacent to the CACGTG motif (Figure 6C). Compared with PS, DM protein also exhibited fewer stabilizing interactions with the DNA sugar-phosphate backbone, consistent with reduced *PHM6* oligo binding by the DM sample in the EMSA. *PHM6* binding by PS complexed with a single Ca^2+^CaM molecule, which interacts with the N-terminal sequence and PCBD2 site, was also modeled (Figure 6D), although its binding was not investigated by EMSA. CaM binding to PS, like the DM mutations, aligned PCBD1 residue K210 with the minor groove, where it interacted with G34 within the CACGTG motif and its complementary nucleobase C33. Residue K186 interacted with C22 in the major groove, and PS/CaM binding was stabilized by additional major and minor groove backbone contacts. Additional modeling of PS binding to *WRKY42* and *PER50* sequences predicted “5-TGGTA-3′ and 5′-GGGGA-3′ recognition sequences, respectively, suggesting a 5′-(G/T)GG(G/T)A-3′ consensus sequence. A logo representation of their frequencies is shown in Figure S6.

## 3. Discussion

Apyrases are located in different subcellular compartments, where they potentially regulate different cellular processes [4]. The pea apyrase PS has been localized in both the ECM [10,13] and in nuclei [9,10]. Its functions in the ECM likely include regulation of [eATP] and downstream purinergic signaling networks, as has been shown for other plant ecto-apyrases [3]. ECM-localized PS activities have also been shown to facilitate Pi mobilization from eATP [13]. However, its functions in the nucleus are poorly understood.

Ectopic expression of PS results in distinct changes in gene expression in Arabidopsis and soybean [12,14], which help to explain the common phenotypes seen in PS plants. These include an expanded root system architecture, enhanced growth and seed yields, increased phosphate uptake, and drought tolerance [11,12,13,14]. The purpose of this study was to explore potential functions of the nuclear-localized PS in transcriptional regulation of yeast and Arabidopsis genes.

A previous study confirmed that a bipartite nuclear localization signal (NLS_BP) in PS protein was required for targeting to plant nuclei [14]. In yeast, the enhanced growth phenotype of a PS-overexpression line was not seen in a line expressing a mutant PS protein in which the NLS_BP sequence was deleted. However, the deleted NLS_BP sequence occurs in the “hinge” region of the enzyme and includes potential DNA-binding residues that we identified in the current study. A deletion in this region would likely impair PS function as a TF even if nuclear targeting was not disrupted. Interestingly, the introduction of the two point mutations into the PCBD1-DM sequence reduced the amount of DM protein in Arabidopsis nuclei, relative to PS [14]. However, lower-affinity binding of DM to dsDNA, rather than reduced nuclear targeting, might also explain reduced DM contents in this organelle, with increased loss of DM occurring during purification of chromatin fractions from nuclei. In yeast co-IP experiments, nuclear localization sequence-binding protein NSR1 was an interacting partner with both PS and DM proteins. NSR1 specifically binds the NLS of histone H2B [36], and its binding to the NLS of these proteins may facilitate their transport into the nucleus. Approximately equal amounts of PS and DM protein were found in chromatin fractions from purified yeast nuclei [14], suggesting that the altered NLS-BP spacer sequence does not significantly affect nuclear import of DM protein in yeast.

In the nucleus, PS could interact with chromatin proteins to influence gene expression in a variety of ways. Its NTPDase activities might locally alter [ATP] and ATP-dependent processes in the nucleus, such as DNA replication, transcription, and RNA processing. Nuclear PS activities or interactions with other chromatin proteins might be regulated by Ca^2+^/CaM binding, as has recently been reported for Calmodulin binding transcription factors (CAMTAs; [37]). CaM, co-purified with PS and other chromatin proteins from nuclei in a yeast PS overexpression line [14], and Ca^2+^/CaM increased the activity of PS purified from pea nuclei by 3-fold [9]. In the present study, modeling of a Ca^2+^/CaM-PS complex with the *PHM6* oligonucleotide showed altered binding site interactions around the Pho4p motif. This might be one way that CaM binding could modulate PS interactions with target sequences. PS protein–protein interactions might also be involved in epigenetic modifications or recruitment of transcription factors to their promoter binding sites, as is suggested by the TF SCL-30 in our co-immunoprecipitation analyses.

We used ChIP-seq to identify PS- and DM-binding sites in promoters of Arabidopsis genes, then integrated these data with gene expression data to identify potentially regulated target genes, as had been performed for yeast genes in a previous study [14]. Further functional studies of PS- and DM-binding to yeast or plant promoters included identification of over-represented motifs for known yeast or plant TF in binding site sequences, and characterization of PS- or DM-binding to selected binding site sequences with enriched motifs using EMSA.

The promoter of yeast gene *PHM6* exhibited a PS binding site with a CACGTG motif for the Pho4p TF that regulates phosphate starvation-responsive genes in the *PHO* regulon [33]. Although *PHM6* is induced in response to P limitation, it appears to function in Pi accumulation only under phosphate surplus conditions [34]. Indeed, PS yeast did not accumulate more Pi than a control line at low [Pi], but exhibited approximately 2-fold higher Pi contents than the control after a short incubation in a medium with higher [Pi] [14]. *PHM6* was one of a dozen genes that were induced by P limitation in the PS line and whose promoters exhibited PS-specific binding (no DM-binding sites), suggesting that PS binding might help regulate P homeostasis in this organism. Functional assays with the *PHM6* promoter supported a PS-specific binding to this sequence, with no significant DM binding. Partial binding to the CACGTG motif was supported by the observed lack of PS binding to an oligo containing a mutated Pho4p site. Structure modeling predicted overlapping PS and DM binding sites involving the Pho4p motif, with off-site binding in the mutated oligo. The reduced number of DM interactions with DNA was consistent with lower DNA-binding affinities for this protein, relative to PS. Pho4p was not detected as a PS or DM binding partner in yeast co-IP assays, so indirect binding of PS or DM complexes with this yeast transcription factor does not explain their interactions with its binding site, although complexes with other proteins in the partially purified PS/DM samples could account for the observed binding data.

An enriched DOF1.5 motif 5′-AAAG-3′ is located in a PS-binding site in the promoter for *WRKY42*, which was induced in PS seedlings. EMSA supported PS-specific binding to this sequence, since some band shifts occurred only with PS-containing samples and were not seen if the DOF1.5 motif was mutated. However, structure modeling predicted binding to a ‘5-TGGTA-3′ sequence adjacent to but not overlapping with the DOF1.5 motif. Under conditions of Pi sufficiency in which the seedlings were grown, WRKY42 binds to the promoter of major Pi transporter *PHT1;1* (At5g43350), increasing its expression [38]. Under Pi sufficiency, PHT1;1 plays the major role in Pi uptake into the root, since other PHT1 transporters are repressed [39]. The potential role of PS in regulating *WRKY42* expression, with downstream effects on expression of known WRKY42 targets like *PHT1;1* and *PHO1* [38], was of particular interest to us because a Pi accumulation phenotype has been reported for PS seedlings growing under Pi sufficiency [13,14]. As in yeast, Pi accumulation does not occur in PS seedlings grown under Pi limitation [14], where *WRKY42* is repressed and the WRKY42 protein is degraded [38], further supporting a possible role for this TF in the Pi accumulation phenotype. However, neither *PHT1;1* nor *PHO1* was DE in PS seedlings [14]. Their regulation by WRKY42 was previously studied in overexpression lines exhibiting 50 to 150-fold higher *WRKY42* expression than WT seedlings [38]. The much lower induction level for *WRKY42* in PS seedlings may be below the response threshold for differential expression of these targets. It is noteworthy that of 74 genes with functionally characterized WRKY42 binding sites (PlantRegMap; https://plantregmap.gao-lab.org/cis-map.php; accessed 12 November 2025), only a single target gene, Scarecrow-like 3 (AT1G08695), was DE in PS seedlings (Table S1). 

We further demonstrated PS-specific binding to a *PER50* promoter sequence containing SHN3 consensus 5′-GCC-3′ box elements. Binding to an oligo with a mutated SHN3 sequence, which overlapped the 5′-GGGGA-3′ binding site predicted by structure modeling, was abolished. Two important functions for plant peroxidases like PER50 are the regulation of H_2_O_2_ metabolism in response to oxidative stress and also the utilization of H_2_O_2_ as a substrate to cross-link proteins and induce lignin formation in the cell wall [28]. The latter processes decrease wall extensibility and growth [29]. Lim et al. [30] showed that suppression of *AtAPY1/2* in the R2-4A line increased H_2_O_2_ production, and cell wall lignification also increased, in parallel with the induction of five class III peroxidases and growth inhibition in Arabidopsis seedlings. In contrast, *AtAPY2* overexpression led to the decreased expression of these peroxidases and increased growth in Arabidopsis seedlings [39]. The PS-specific induction of *PER50* was at the statistical cutoff for differential gene expression (FC ≥ 1.5), and its contribution to the enhanced growth phenotype of PS seedlings is unclear. It was not DE in the R2-4A line [30]. It is interesting to note, however, that nine other *PER* genes were DE in PS seedlings but not in DM seedlings ([14], Table S6). Two of these peroxidase genes (*PER15*, *PER53*) were strongly induced in the R2-4A line [30] but were repressed in PS seedlings, which is consistent with the proposed model for apyrase-regulated peroxidase expression and growth regulation [4].

PS-specific binding to the enriched SHN3 motif is also interesting in that ectopic expression of PS in soybean induced a large number of genes involved in wax and cuticle synthesis [12]. This resulted in a reduced cuticle permeability that contributed to a drought-tolerance phenotype of these plants, which were not experiencing water limitation. SHN3 belongs to a TF clade whose members activate genes involved in wax synthesis and cuticle formation in Arabidopsis [27]. None of the SHN1-3 TF were DE in PS soybean; thus, PS binding to SHN3 elements may have enhanced cuticle development, allowing the plant to better adapt to actual drought stress. This may help to explain the significantly increased seed yields in field studies with PS soybean [12]. In PS Arabidopsis seedlings, *SHN2* was induced 1.8-fold, and two downstream targets, cutin synthase (*CUS2*) and very-long chain fatty acyl CoA reductase 3 (*CER4*), were induced 1.6- and 1.5-fold, respectively ([14]; RNA-seq dataset). Interestingly, a nearly identical expression profile for *SHN2*, *CUS2,* and *CER4* was also seen in an *AtAPY1* overexpression line in the Ws background (unpublished data), suggesting that induction of wax synthesis and cuticle formation genes may be a common response to overexpression of an “apyrase 1-type” enzyme.

We used AlphaFold3 to model PS and DM binding to both test and mutant sequences of *PHM6*, mapping specific binding site interactions. These modeling studies (1) identified specific PS/DM residues that may be involved in DNA-binding, with the PCBD1 helix playing an important role in interactions with major groove nucleobases; (2) supported sequence-specific binding for PS to a conserved 5′-(G/T)GG(G/T)A-3′ motif in three different binding site sequences, which overlapped or was adjacent to enriched TF motifs in each binding site; (3) elucidated the potential structural basis for observed differences in PS and DM binding to DNA in EMSA, and (4) showed that CaM binding to PS alters binding site interactions around the Pho4p motif in the *PHM6* oligo. Although this tool is trained using static molecular structures in the Protein Data Bank and cannot simulate buffer conditions used for EMSA, it is capable of highly accurate prediction of protein-DNA complexes. Future modeling using molecular dynamics simulations incorporating the EMSA binding conditions will be helpful in refining binding site predictions.

In both yeast [14] and Arabidopsis, there is relatively little overlap between PS- and DM-binding sites, the over-represented TF motifs, or the expression profiles for potentially regulated target genes, suggesting that PS and DM may regulate different transcriptional networks in each organism. More definitive evidence, such as transcriptional activation assays of putative target genes, using wild-type and mutated-motif promoter fragments and highly purified PS and DM proteins, will be needed to understand the potential role of PS in transcriptional regulation.

Future experiments should include functional verification of DM-specific binding site sequences, such as the MYB-related 5′-ATAAG-3′ sequence in the MYBS1 and SRM1 promoters. They should also examine conserved core sequence elements shared by PS- and DM-binding site overlaps. For example, it is interesting that the 5′-AAG-3′ element found in both the PS-specific DOF1.5 and DM-specific MYB-related TF motifs in Arabidopsis promoters is also contained within the yeast Azf1p motif 5′- AAAAGAAA-3′, which was the most enriched motif in both PS- and DM-specific binding sites in yeast promoters [14]. Enriched TF motifs within PS binding sites exhibit considerable sequence diversity. If these motifs are involved in PS binding, as is suggested by the EMSA data, such binding likely occurs with low sequence specificity, perhaps in complexes with other DNA-binding proteins, such as those identified in co-IP assays. However, the predicted 5′-(G/T)GG(G/T)A-3′ consensus motif derived from modeling studies with the three unrelated promoter sequences used in EMSA suggests a degree of sequence specificity in PS binding. This consensus motif is found within 7% of DOF motifs and 17% of SHN3 motifs within PS binding sites in Table S3. Thus, for a significant number of these genes, PS may function as a DNA-binding regulatory protein, possibly activating or repressing expression of DOF- or SHN3-regulated genes.

## 4. Conclusions

In the present study, we have shown, for the first time, that a plant apyrase may function as a DNA-binding regulatory protein, extending its known enzymatic functions in the Golgi or the extracellular matrix. The nuclear localization of ectopically expressed PS, functional characterization of PS binding to both yeast and Arabidopsis promoter sequences identified in ChIP-seq assays, and structure modeling of these interactions support this novel function. Mutations in a potential calmodulin-binding site PCBD1 of PS, producing the DM protein, altered binding site interactions *in vivo* (ChIP-seq) and *in vitro* (EMSA), with the latter being supported by structure modeling. Importantly, this altered binding was correlated with differences in genome-wide gene expression profiles and related phenotypic characteristics, such as drought tolerance and increased phosphate acquisition in yeast and plants [11,12,14]. Further studies with other plant apyrases will be needed to confirm their potential roles in transcriptional regulation.

## 5. Materials and Methods

### 5.1. Plant Materials and Growth Conditions

*Arabidopsis thaliana* Columbia-0 (Col-0) ecotype was used as the wild-type control. Transgenic lines overexpressing the PS and DM proteins were generated previously by introducing constructs encoding either PS or a DM variant, which contained two amino acid substitutions within a potential calmodulin binding site and an overlapping nuclear localization signal [14]. Homozygous T3 lines were selected for kanamycin resistance, and transgene expression was confirmed by RT-PCR and Western blot analyses. All seedlings were grown for 7 days on half-strength Murashige and Skoog (1/2x MS) agar plates supplemented with 1% sucrose under) under a full-spectrum light intensity of 150 mmol m^−2^ s^−1^ at 22 °C.

### 5.2. Assay for Differential Binding of PS and DM to dsDNA-Cellulose

All operations were carried out at 4 °C. Partially purified PS or DM protein fractions (4.8 µg protein in 20 mM Hepes, pH 7.5) were applied to a dsDNA-cellulose column equilibrated with at least 10 bed volumes of Wash Buffer (20 mM Hepes, pH 7.5, 1 mM EDTA, 1mM Pefabloc protease inhibitor). Purified PS/DM was diluted to 160 ng/µL with 20 mM Hepes (pH 7.5) immediately before being loaded onto the column. After application of PS or DM, the column was washed with 3 bed volumes of Wash Buffer, followed by Wash Buffer containing increasing NaCl concentrations (0.05 M, 0.1 M, 0.3 M, and 0.6 M). As negative controls, PS and DM samples were run on cellulose-only control columns, and ovalbumin (6 µg) was run on a dsDNA-cellulose column. Eluted protein fractions were further concentrated and then analyzed by SDS-PAGE immunoblot assays using the highly specific monoclonal antibody 8B6. Ovalbumin was detected by Coomassie Blue staining.

### 5.3. ChIP-Seq Analyses

#### 5.3.1. Construction and Verification of 4Myc-Tagged PS and DM Overexpression Lines

*PS*- and *DM*-coding sequences were amplified using relevant primers (Table S7) and cloned into pENTR vectors, respectively. Then, pENTR-PS and pENTR-DM vectors were recombined into the pGWB17 plant expression vector (NovoPro #V005425) behind a 35S promoter and in-frame with an N-terminal 4-Myc tag using LR Clonase II. Recombinant vectors were verified to have no coding errors by sequencing, and then were transformed into the Col-0 background. T1 transformants were identified using c-Myc antibody (9B11, Cell signaling Cat# 2276S) to confirm PS expression. T2 and T3 homozygous transformants were selected in the presence of hygromycin antibiotic screening.

#### 5.3.2. Chromatin Immunoprecipitation (ChIP) and Sequencing Library Preparation

Seven-day-old seedlings were harvested, crosslinked with 1% formaldehyde under vacuum for 10 min, and quenched with 0.125 M glycine. Nuclei were isolated from the seedlings, as previously described [14], and chromatin was fragmented to an average size of 200–500 bp by sonication. Immunoprecipitation of 4Myc-tagged PS and DM proteins was performed using EZview Red Anti-c-Myc Affinity Gel (Cat# E6654, Sigma-Aldrich, St. Louis, MO, USA) for one hour in the dark at 4 °C. Input and ChIP DNA were purified, as was reported for yeast [14], and used for library construction with a NEBNext Ultra II DNA Library Prep Kit, following the manufacturer’s protocol.

#### 5.3.3. ChIP-Seq Data Processing, Peak Calling, and Functional Annotation

DNA samples were sequenced by the Genome Sequencing and Analysis Facility (GSAF) at the University of Texas at Austin (Illumina NovaSeq SP, PE150 reads). ChIP-seq data were deposited in the NCBI Sequence Read Archive (accession #PRJNA1268411). fastpXpress, a custom batch fastp script implemented in a Google Colab notebook (https://github.com/rdslocum/fastpXpress; accessed on 12 November 2025), was used to remove adapters and quality trim the initial sequence reads. ChIP-seq analyses were carried out using the CLC Genomics Workbench 10.0.1 platform (Qiagen, Hilden, Germany). An average of 33.7 million reads from two independent experiments were combined and uniquely mapped to the TAIR10 reference genome with an average 38.8x genome coverage. Putative PS- and DM-binding sites were identified (“peak calling”) using input controls and overlap analyses, comparing peak profiles in WT versus *PS* and *DM* overexpression lines. Quality control metrics for these assays are summarized in Table S8. Peaks were annotated using expanded Araport11 annotation based upon the AtRTD3 reference transcriptome [40]. High-confidence binding sites (peak shape scores ≥ 2.0 and *p* ≤ 0.05) located 1–1500 bp upstream from the TSS of genes on both sense (+) and antisense (−) strands were identified. Further overlap analyses identified unique PS- and DM-specific binding sites, and a subset of overlapping PS- and DM-binding sites. Gene Ontology (GO) BioProcess enrichment analysis for potential target genes was performed using the PANTHER 19.0 database (https://www.pantherdb.org/).

#### 5.3.4. PS and DM Binding Site Motif Enrichment Analyses

Identification of conserved *cis*-regulatory elements, via motif enrichment analysis, was conducted using the MEME Suite v5.5.7 SEA tool (https://meme-suite.org/meme/tools/sea; accessed on 12 November 2025) with the DAP-seq database of 872 Arabidopsis transcription factor-binding motifs [41]. Significantly enriched motifs (≥2-fold enrichment, *p* ≤ 0.01) were identified in PS- or DM-specific binding site sequences within promoters of genes that were also differentially expressed only in PS- or DM-overexpressing Arabidopsis seedlings, respectively.

### 5.4. Functional Characterization of PS and DM Binding Sites Using Electrophoretic Mobility Shift Assays

High confidence PS-binding sites located within the promoters of genes that were differentially expressed only in PS yeast, or in PS seedlings, were identified from ChIP-seq analyses. Binding sites exhibiting enriched motifs with high sequence similarities to known TF binding sites that were also located approximately in the center of the binding site were filtered from the larger dataset. The potential functional significance of those binding events was further characterized *in vitro* using electrophoretic mobility shift assays (EMSA). The Pho4p motif CACGTG in the yeast *PHM6* promoter, the Dof1.5 motif AAAAGAAA in the Arabidopsis *WRKY42* promoter, and the SHN3 consensus GCC box motif in the Arabidopsis *PER50* promoter were selected for EMSA validation.

#### 5.4.1. Oligonucleotide Probe Preparation

Double-stranded DNA probes, comprising approximately 30 bp upstream and 30 bp downstream of the central motif, were used in EMSA. Single-stranded forward and reverse complement oligonucleotides were synthesized by Integrated DNA Technologies (IDT, Coralville, IA, USA). Biotin labeling was performed at the 3′ end of each strand using the Biotin 3′- End DNA Labeling Kit (Thermo Scientific #89818) following the manufacturer’s protocol. For annealing, equimolar amounts of labeled forward and reverse strands were mixed, denatured at 90 °C for 1 min, and slowly cooled to room temperature, followed by incubation at the melting temperature for 30 min. Unlabeled competitor probes were prepared in parallel using the same annealing steps. For each candidate binding site, a corresponding mutated oligonucleotide probe was synthesized by altering the core motif sequence. The mutated regions were designed to disrupt potential binding without introducing matches to other known TF motifs in the dataset. Motif scanning analysis confirmed that the mutated elements did not overlap with any enriched motifs identified in PS binding sites.

#### 5.4.2. EMSA Procedure

EMSA reactions were performed using the LightShift^®^ Chemiluminescent EMSA Kit (Thermo Scientific #20148), following the manufacturer’s protocol. Recombinant PS and DM proteins were purified as described in the protein expression and purification section. Binding reactions were assembled in 20 µL volumes containing 10 nmol biotin-labeled probe, 200 ng (4.2 pmol) recombinant PS or DM protein, and binding buffer (10 mM Tris, pH 7.5, 50 mM KCl, 1 mM DTT, 1 µg/µL poly dI·dC). For competition assays, a 200-fold molar excess (2 µmol) of unlabeled competitor probe was included. Reactions were incubated at room temperature for 30 min and resolved on 6% native polyacrylamide gels in 0.5x TBE buffer at 100 V. DNA-protein complexes were transferred to a nylon membrane, cross-linked, and detected using the chemiluminescent detection reagents provided in the kit. Positive control reactions were performed using Biotin-EBNA Control DNA and EBNA extract supplied with the kit.

### 5.5. Purification of PS and DM Proteins

The purified PS and DM were partially purified from extracts of PS- or DM-overexpressing strains [14]. Yeast cells (~2 g wet weight) were homogenized with a Kinematica polytron homogenizer in 10 mL of homogenization buffer (50 mM Tris-Cl pH 7.4, 150 mM NaCl, 0.2% NP-40, 10% Glycerol, 1 mM PMSF protease inhibitor) then centrifuged at 13,000× *g* for 10 min at 4 °C. After filtering the supernatant through a 0.45 µm filter, proteins in the supernatant were enriched by ammonium sulfate precipitation (20%, then 80% *w*/*v*). The pellet was washed and dissolved in resuspension buffer (60 mM Hepes pH 7.5, 3 mM MgCl_2_, 1 mM Pefabloc, and 10 mM 2-mercaptoethanol), then the solution was dialyzed against 10 mM Tris-HCl and 20 mM NaCl at pH 8.0 buffer for 4 h. The sample was then concentrated using an Amicon concentrator. The final protein concentration was confirmed by using the Bradford method. PS and DM purity was ~1% in these protein preparations, which contained approximately equal amounts of PS and DM protein, as estimated by SDS-PAGE immunoblot analyses using the highly specific monoclonal antibody 8B6 and ≥95% purity PS protein as the standard for densitometric analysis (Figure S7). The antibody and purified PS protein were commercially prepared by GenScript (Piscataway, NJ, USA).

### 5.6. Co-IP for Yeast and Arabidopsis

#### 5.6.1. Yeast Protein Preparation

Yeast NS219 transformed with pYES2 vector alone (empty vector (EV) control), pYES2-psNTP9, or pYES2-psNTP9-DM were initially cultured in 4 mL liquid yeast nitrogen base (uracil dropoff) with glucose, pH 5.2. The culture was grown at 30 °C for 48 h on a 250 rpm shaker. After 48 h, the cultures were inoculated (starting OD_660_ = 0.03) into 100 mL of induction medium containing 2% galactose and grown to a final OD_600_ = 0.6–0.8. Cells were harvested by centrifugation at 3000× *g* for 10 min at 4 °C. The cell pellet was dissolved in YeastBusterTM Reagent and THP Solution with 25 U Benzonase^®^ Nuclease and 500 µL protease inhibitors and incubated for 20 min at room temperature. Insoluble cell debris was removed by centrifugation at 16,000× *g* for 20 min at 4 °C, and the supernatant was used in the following immunoprecipitation step.

#### 5.6.2. Arabidopsis Protein Preparation

Seven-day-old Arabidopsis seedlings (~5 g) of ecotype Columbia (CS907), or PS-4Myc, or DM-4Myc transgenic lines, were collected and ground in liquid nitrogen. Total protein was solubilized in extraction buffer (50 mM Tris, pH 7.5, 150 mM NaCl, 1 mM EDTA, 0.1% Tween-20, 0.25 mM DTT, 1× protease inhibitor cocktail, 1 mM PMSF). The extracts were centrifuged at 20,000× *g* for 15 min, and the supernatant was used in the immunoprecipitation step.

#### 5.6.3. Co-Immunoprecipitation

Yeast or Arabidopsis seedling extracts were incubated with EZview Red Anti-c-Myc Affinity Gel (Cat# E6654, Sigma-Aldrich) for one hour in the dark at 4 °C. Resin was washed with 1 mL of TBS-T three times for 10 min each. Myc-tagged proteins were eluted with 50 µL 0.2 M Glycine pH 2.6, then the eluate was immediately neutralized with 120 µL pH 9.4 Tris-HCl. SDS-PAGE immunoblot analyses with Anti-Myc antibody were used to detect immunoprecipitated Myc-tagged PS or DM proteins.

#### 5.6.4. Mass Spectrometry Analysis

Co-IP preparations were submitted for mass spectrometry analysis at the Biological Mass Spectrometry Facility, UT Austin Center for Biomedical Research Support (RRID:SCR_021728). Prior to LC-MS analysis, proteins were reduced, alkylated, and digested with trypsin, and the resulting peptides were either manually or robotically desalted using Millipore U-C18 ZipTip pipette tips following the manufacturer’s protocol. Data were acquired using an Orbitrap Fusion Tribrid mass spectrometer equipped with an ultra-high-pressure Ultimate 3000 RSLC nanosystem (Thermo Scientific, Waltham, MA, USA). Raw data files were analyzed using MaxQuant version 2.0.3.0 [42]. The LFQ protein quantification strategy [43], match between runs, and a 1% false discovery rate at both the peptide spectrum match and the protein level were employed.

#### 5.6.5. Identification of Potential PS and DM Co-IPs

We considered co-IPs in the control extracts of yeast (pYES2 empty vector) or Arabidopsis seedlings (no ectopic expression of PS or DM) to be “false-positives”. If the same co-IPs occurred in PS or DM assays, we did not consider them further. We filtered the list of potential co-IPs (87 Arabidopsis and 51 yeast proteins) using annotated lists of yeast and Arabidopsis TF (SGD, https://www.yeastgenome.org/; AtTFDB, https://agris-knowledgebase.org/AtTFDB; both URLs accessed on 12 November 2025) and chromatin proteins, downloaded from the Uniprot database (https://www.uniprot.org/; accessed on 12 November 2025), to produce a realistic set of co-IPs that may facilitate DNA binding in complexes with PS or DM in EMSA (Table S5).

### 5.7. Gene Expression Analyses

Gene expression analyses were performed using 7-day-old Arabidopsis seedlings that overexpress PS (PS2 line) or DM (DM4 line). Total RNA concentration and purity were measured using Thermo Scientific NanoDrop 1000 spectrophotometer. Approximately 1 µg of total RNA treated with Amplification Grade DNase I was reverse transcribed using a High-Capacity cDNA Reverse Transcription kit (Applied Biosystems/Thermo Fisher, Waltham, MA, USA).

qRT-PCR was performed using the Life Technologies/AB Biosystems Quant 7 Real-Time PCR System. Power SYBR Green PCR Master Mix (Life Technologies/AB Biosystems, Waltham, MA, USA) was used for RT-qPCR reactions. The qRT-PCR conditions used were hold stage: 50 °C, 2 min, and 95 °C, 10 min; PCR stage: 40 cycles of 95 °C, 15 s, and 60 °C, 1 min. Melting curves generated from machine dissociation conditions were used to identify primer dimers and multiple targets. The primer sequences used are listed in Table S7**.** For each biological replicate, three technical triplicates with ±0.5 CT were used for qRT-PCR analysis. The relative gene expression was calculated by the 2^−ΔΔCT^ method [44]. *Actin1* was used as the reference gene in the Arabidopsis qRT-PCR. Statistical significance between treatments was calculated by Student’s *t* test, using ΔCT for three biological replicates.

Transcriptome-wide gene expression analyses (RNA-seq) and qRT-PCR validation of expression for selected genes were previously reported [14]. RNA-seq data were deposited in the NCBI Sequence Read Archive (accession #PRJNA449074).

### 5.8. Structural Studies of PS and DM

AlphaFold3 was accessed via its server (https://alphafoldserver.com/; accessed on 12 November 2025). Sequences used in modeling studies: mature PS (NP_001414507.1; Q22-V455, N-terminal 21 residues deleted), DM protein (mature PS with S208L and P216R mutations), Arabidopsis Calmodulin 1 (NP_198594.1), sense and anti-sense oligonucleotides for *PHM6*, *WRKY42*, and *PER50* binding sites (Table S7). Residue numbering for the full-length precursor PS or DM proteins was used. The CaM1 binding model included Ca^2+^. All models included K+ and Cl-, since the EMSA binding buffer contained 50 mM KCl.

## Figures and Tables

**Table 1:** Frequency of statistically enriched TF motifs in PS- or DM-specific binding sites within promoters of Arabidopsis genes. Motif enrichment analyses were carried out using the MEME 5.7.5 suite SEA tool with the DAP-seq database of 872 Arabidopsis transcription factor binding motifs (https://meme-suite.org/meme/tools/sea, accessed 12 November 2025).

Binding Site Specificity	TF Family (DAP Database)	TF Motif	TF Motif Consensus Sequence	Binding Sites with Motif	Percent Total Sites with a TF Motif	*p*-Value (FDR-Corrected)
PS	ABI3VP1	VRN1	VWKTTTTTTTTTTTTTTKB	35	15.56	2.67 × 10^−4^
PS	AP2EREBP	SHN3	WCCDCCGCCRCCDCCGCCGCC	11	4.89	1.12 × 10^−2^
PS	LOBAS2	LOB	WCCGCCGCCDYCKCCGCCGCH	22	9.78	4.06 × 10^−3^
PS	C2H2	JKD, IDD10	WWWWTTTTTGTCKTTTTSTD	15	6.67	9.60 × 10^−3^
PS	Homeobox	ATHB53	HCAATAATTGD	37	16.44	1.11 × 10^−2^
PS		ATHB20, GL2	HYAATAATTRA	37	16.44	1.11 × 10^−2^
PS		ATHB1, HAT5	HYAATAATTRW	30	13.33	1.13 × 10^−2^
PS	BBRBPC	BPC1	CTCTYTCTCTCTCTC	66	29.33	7.38 × 10^−3^
PS	C2H2	TF3A	CYTCCTCCTCCTCCTCCTC	26	11.56	5.66 × 10^−3^
PS	ND	FRS9	CTCTCTCTCTCTCTCTCTCTC	24	10.67	3.50 × 10^−3^
PS	WRKY	WRKY27	ANCGTTGACTTTT	26	11.56	1.47 × 10^−3^
PS		WRKY7	DNCGTTGACTTTTT	32	14.22	3.30 × 10^−3^
DM	bZIP	ABI5	DDTGRWSACGTGGCA	10	9.35	5.99 × 10^−3^
DM	MYB related	MYBS1	HWWAWYCTTATCYWH	13	12.15	1.09 × 10^−2^
DM		MYBS1	DWWDWRGATAAGRTT	10	9.35	5.99 × 10^−3^
DM		SRM1	DWWDWRGATAAGR	10	9.35	5.99 × 10^−3^
DM		SRM1	YCTTATCYWHW	11	10.28	5.01 × 10^−4^
PS	C2C2dof	DOF1.1	TTTTYACTTTTTYTTTTTTTTTTTTTW	59	26.22	7.71 × 10^−5^
PS		DOF1.5, COG1	RWAAAAADDAAAAAGTRAAAA	60	26.67	9.20 × 10^−3^
PS		DOF1.7, Adof1	AAAAAVAAAAAGTARAAAAWR	22	9.78	1.86 × 10^−3^
PS		DOF5.1	HWTTWACTTTTTBDHTTWW	30	13.33	1.13 × 10^−2^
PS		DOF5.6, HCA2	RAAAAAADVAAAAAGKWAAWA	40	17.78	1.60 × 10^−3^
DM		DOF3.2	KTWACTTTTTNNYTTTTTT	17	15.89	3.72 × 10^−3^
DM		DOF5.8	TTTWTTTACTTTTTBYTTTTT	17	15.89	3.72 × 10^−3^

## Data Availability

The Arabidopsis ChIP-seq and RNA-seq sequence reads files are available in the NCBI Sequence Reads Archive (accession #PRJNA1268411 and #PRJNA449074, respectively). The custom sequence reads preprocessing script fastpXpress is available at (https://github.com/rdslocum/fastpXpress; accessed on 12 November 2025).

## Supplementary Materials

The following supporting information can be downloaded at: https://www.mdpi.com/article/10.3390/plants14223514/s1. Figure S1: Venn diagram showing overlap between sets of potentially regulated Arabidopsis genes with PS- or DM-specific binding sites in their promoters.; Figure S2: PS-specific binding site in the yeast *PHM6* gene promoter and test and mutated oligo sequences used in functional testing of the binding site with EMSA.; Figure S3: PS-specific binding site in the Arabidopsis *WRKY42* gene promoter and test and mutated oligo sequences used in functional testing of the binding site with EMSA; Figure S4: PS-specific binding site in the Arabidopsis *PER50* gene promoter and test and mutated oligo sequences used in functional testing of the binding site with EMSA; Figure S5: Predicted PS binding sites in yeast (*PHM6*) and Arabidopsis (*WRKY42*, *PER50*) promoter sequences used in EMSA; Figure S6: AlphaFold3-predicted interactions of mature PS or DM proteins with the yeast *PHM6* promoter binding site.; Figure S7: Purification of PS and DM proteins for use in EMSA.; Table S1: PS- and DM-specific binding sites in promoters of potential target genes and expression patterns for those genes in PS- and DM-overexpressing Arabidopsis seedlings; Table S2: Differentially expressed genes enriched specifically in an Arabidopsis DM-overexpression line are involved in abscisic acid (ABA) signaling or responses to ABA (BioProcess categories GO:0009737; GO:0009269).; Table S3: PS-specific binding sites and enriched transcription factor motifs in promoters of genes that are differentially expressed only in PS Arabidopsis seedlings (NOTE: This is a separate Excel file.).; Table S4: qRT-PCR validation of *WRKY42* and *PER50* expression in RNA-seq datasets.; Table S5: Chromatin proteins or transcription factors that interact with 4Myc-tagged Arabidopsis PS or DM proteins, or 13Myc-tagged yeast PS or DM proteins, in co-IP assays.; Table S6: Expression profiles for peroxidase genes regulated in response to ectopic *PS* or *DM* expression, or suppression of *AtAPY1/2* in Arabidopsis seedlings.; Table S7: PCR primers and oligos used in this study.; Table S8: Quality control metrics for the Arabidopsis ChIP-seq assay.

## Abbreviations

The following abbreviations are used in this manuscript:

NTPDase	nucleoside triphosphate diphosphohydrolase
eATP	extracellular ATP
CaM	calmodulin
PS	pea apyrase psNTP9
DM	“double mutation” version of PS
DEG	differentially expressed genes
TF	transcription factor
EMSA	electrophoretic mobility shift assay
NLS_BP	bipartite nuclear localization signal
PCBD1	potential calmodulin-binding domain 1
