# Supplementary material for: Chromatin-Associated Pea Apyrase psNTP9 Function as a DNA-Binding Regulatory Protein in Yeast and Arabidopsis
Source: Plants (Basel). 2025 Nov 18;14(22):3514. doi: 10.3390/plants14223514 (PMC12656381; doi:10.3390/plants14223514)
Supplement: Supplementary file 1 [file plants-14-03514-s001.zip › Supplementary Figures and Tables.pdf]

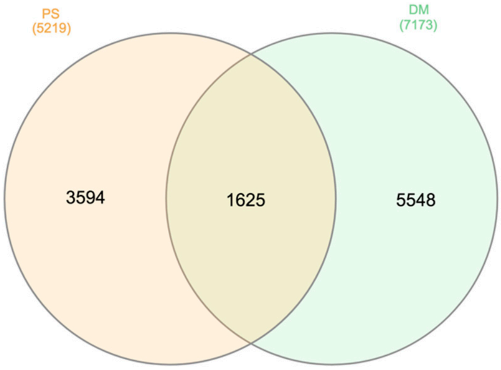

Figure S1. Venn diagram showing overlap between sets of potentially-regulated Arabidopsis genes with PS- or DM-specific binding sites in their promoters.

A

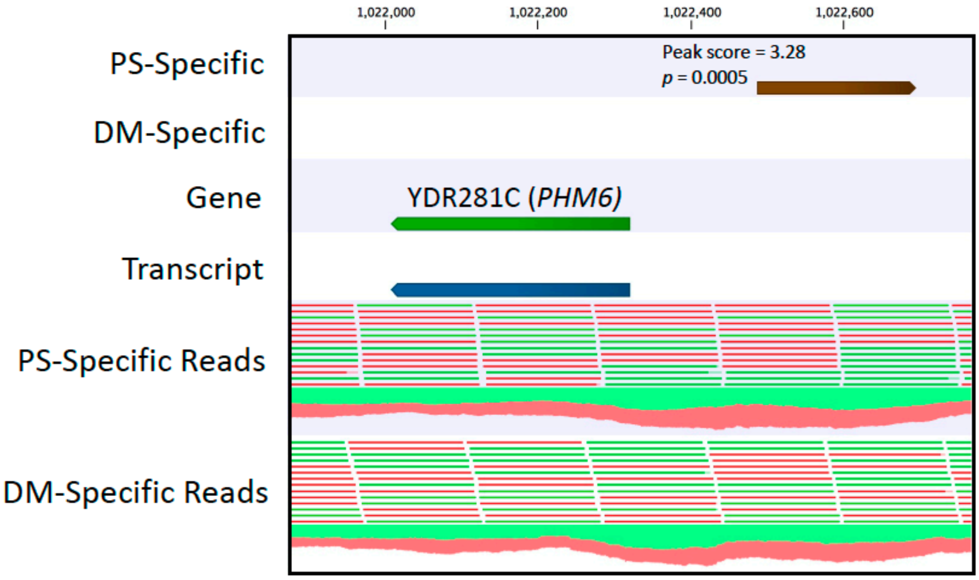

B

Pho4p Consensus: **CACGTG**

Test oligo (66 bp):
5′-ATAATGTTACGCAGTTCCGAGCTTCGCTGA**CACGTG**GGAGGTGGGATATAATATTTG**CACGGG**TTC-3′

Mutated oligo (66 bp):
5′-ATAATGTTACGCAGTTCCGAGCTTCGCTGAGGGCCCGGAGGTGGGATATAATATTTGCCCCCCTTC-3′

Figure S2. PS-specific binding site in the yeast *PHM6* gene promoter and oligonucleotide sequences used in functional testing of the binding site with EMSA. A) Genome browser view of PS- and DM-specific binding sites, relative to the yeast *PHM6* gene and its transcripts on chromosome IV. Distribution of mapped reads (green, sense; red, anti-sense) are indicated. A single PS binding site is located within the *PHM6* promoter. The peak shape score and *p*-value for the PS-specific peak are shown. **B**) DNA sequences of test and mutated oligos used in EMSA with PS and DM proteins. The oligos are comprised of 30 bp of upstream and downstream sequences around the centrally-located Pho4p CACGTG motif, which was changed to GGGCCC in the mutated oligo. A similar 3′-end CACGGG motif was also mutated to CCCCCC to minimize potential PS binding to this sequence.

A

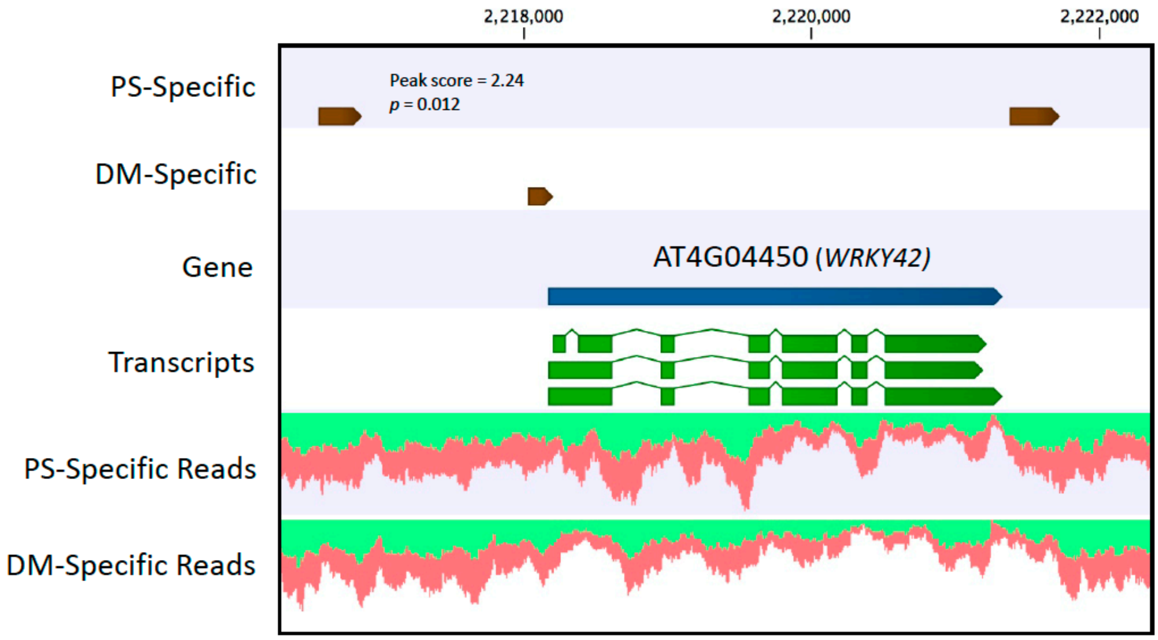

B

Dof1.5 Consensus (5'-AA[AG]G-3' core):

RWAAAAADDAAAAAGTRAAAA

Test oligo (70 bp):

5'-AGTTATTTTAACTAATAAACAGATA**AAAAGAAATAAAAAGTACTAA**ATTGGTAAATTATCGTAGTATCAT-3'

Mutated oligo (70 bp):

5'-AGTTATTTTAACTAATAAACAGATA**AAAAGAAATA**GCCTAC**ACTAA**ATTGGTAAATTATCGTAGTATCAT-3'

Figure S3. PS-specific binding site in the Arabidopsis *WRKY42* gene promoter and oligonucleotide sequences used in functional testing of the binding site with EMSA. Genome browser view of PS- and DM-specific binding sites, relative to the Arabidopsis *WRKY42* gene and its transcripts. Distribution of mapped reads (green, sense; red, anti-sense) are indicated. A single PS binding site is located within the WRKY promoter. The peak shape score and *p*-value for the PS-specific peak are shown. A DM-specific binding site spans the TSS of this gene but *WRKY42* is DE only in PS seedlings. **B**) DNA sequences of test and mutated oligos used in EMSA with PS and DM proteins. The 70 bp oligos are comprised of upstream and downstream sequences around the centrally-located Dof1.5 motif, whose core sequence was changed in the mutated oligo.

A

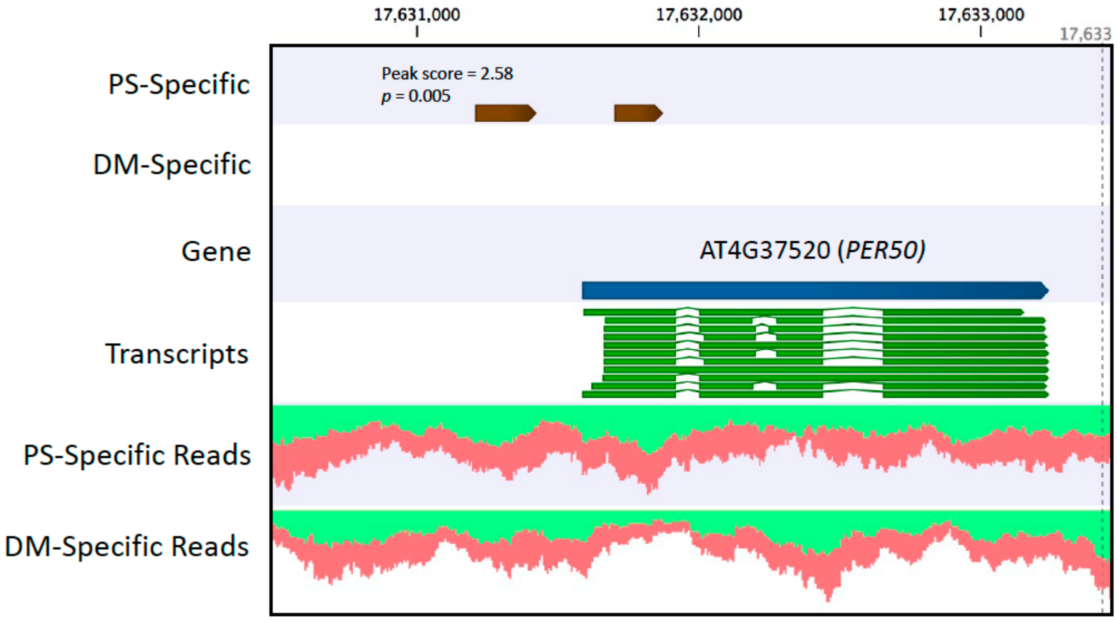

B

SHN3 Consensus (GCC Box element):

WCCDCCGCCRCCDCCGCCGCC

Test oligo (70 bp):

5′-TTCACTTTTCAACTTATCCCTCTCAATCCCCACCACCACCACTCTCTATTCCCTCATATAGATAGATCAT-3′

Mutated oligo (70 bp):

5′-TTCACTTTTCAACTTATCCCTCTCAATCCCCAGTCCTATGACTCTCTATTCCCTCATATAGATAGATCAT-3′

Figure S4. PS-specific binding site in the Arabidopsis *PER50* gene promoter and oligonucleotide sequences used in functional testing of the binding site with EMSA. Genome browser view of PS- and DM-specific binding sites, relative to the Arabidopsis *PER50* gene and its transcripts. Distribution of mapped reads (green, sense; red, anti-sense) are indicated. A single PS binding site is located within the *PER50* promoter and an additional PS binding site is located within exon 1. The peak shape score and *p*-value for the PS-specific peak are shown. **B**) DNA sequences of test and mutated oligos used in EMSA with PS and DM proteins. The 70 bp oligos are comprised of upstream and downstream sequences around the centrally-located SHN3 motif, whose conserved core sequence was changed in the mutated oligo.

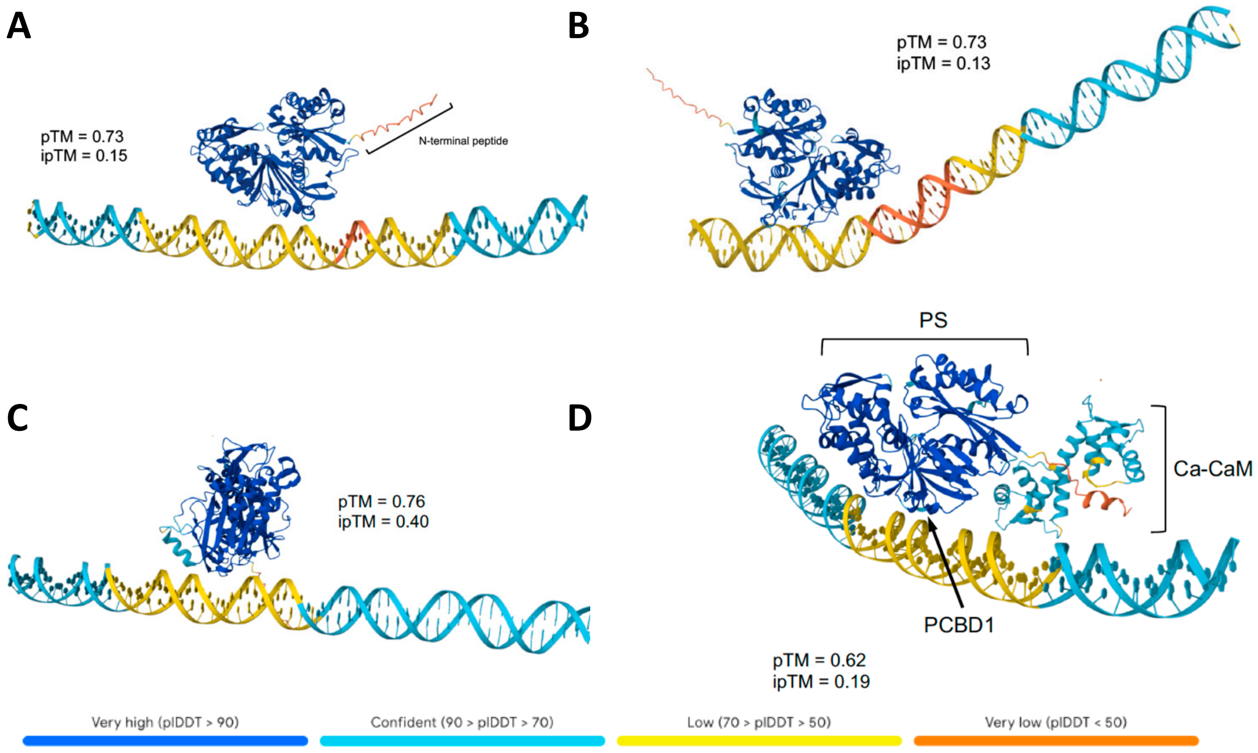

Figure S5. AlphaFold3-predicted interactions of mature PS or DM proteins with the yeast *PHM6* promoter binding site. PS binding to the test oligo (**A**) versus mutant oligo (**B**) sequences. DM binding to the test oligo (**C**). Binding of PS/Ca^{2+}CaM complex to the test oligo (**D**). Confidence scores for local structure predictions are indicated in the pLDDT scale.

PHM6	ATAATGTTACGCAGTTCCGAGCTTCGCTGACACGTGGGAGGTGGGATATAATATTTGCACGGGTTC
WRKY42	AGTTATTTTAACTAATAAACAGATAAAAAGAAATAAAAAGTACTAAATTGGTAAATTATCGTAGTATCAT
PER50	ATGATCTATCTATATGAGGGAATAGAGAGTGGTGGTGGTGGGGATTGAGAGGGATAAGTTGAAAAGTGAA

PHM6	TGGGA
WRKY42	TGGTA
PER50	GGGGA

Consensus: (G/T)GG(G/T)A

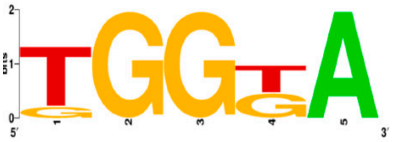

Figure S6. Predicted PS binding sites in yeast (*PHM6*) and Arabidopsis (*WRKY42, PER50*) promoter sequences used in EMSA. Overrepresented TF binding site motifs are indicated in red. Binding sites for mature PS protein (underlined, bold) were predicted using AlphaFold3 to model PS interactions with the oligos. Aligned binding site sequences and the consensus sequence are shown below. *PER50* antisense sequence and sense sequences for *PHM6* and *WRKY42* are shown in 5' → 3' orientation. Relative frequencies of each motif in PS-specific binding sites in promoters of Arabidopsis genes that are also DE only in seedlings that ectopically express PS is represented by the logo (Weblogo3; https://weblogo.berkeley.edu/logo.cgi).

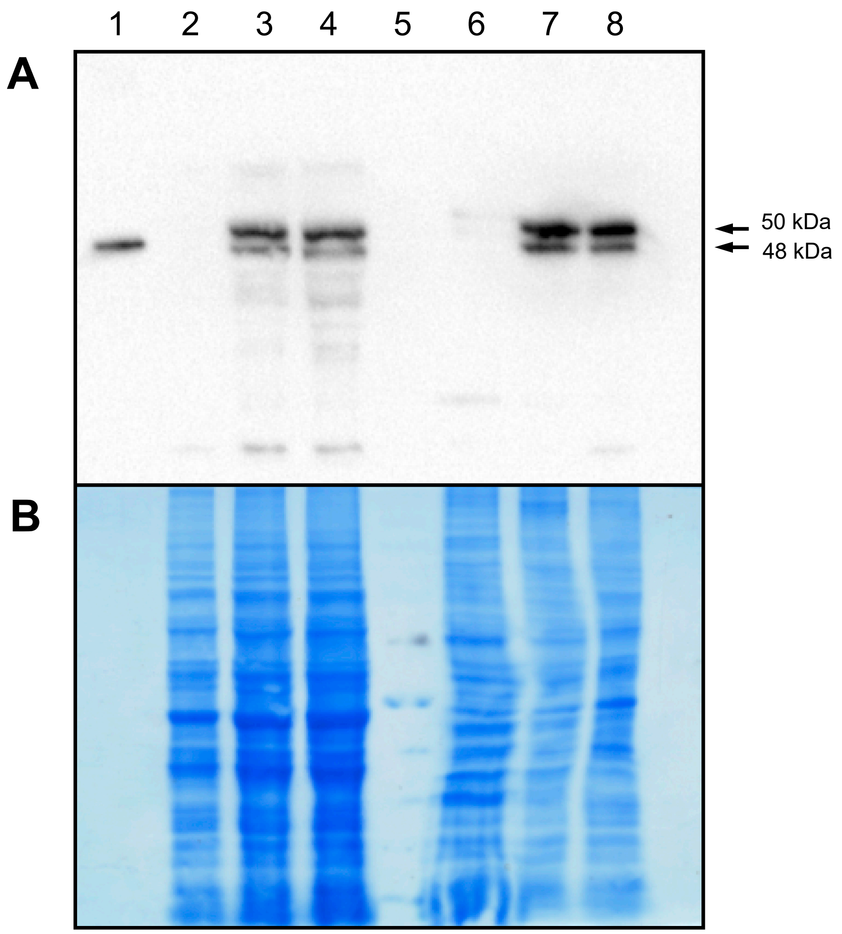

Figure S7. Purification of PS and DM proteins for use in EMSA. Proteins were partially-purified from yeast, as described in the methods section. Purified 48 kDa mature PS protein (20 ng; NTP9-30a) was loaded in lane 1. The initial 20% (w/v) ammonium sulfate (AS) fractions from the pYES2 empty vector control (lane 2) or PS- or DM-enriched proteins (lanes 3 and 4, respectively) were further fractionated with 80% (w/v) AS. 80% AS cuts of pYES2, PS and DM fractions (lanes 6-8), and MW markers (lane 5) were separated by SDS-PAGE gel then blotted (A). Remaining proteins in the gel were visualized by InstantBlue staining (B). The blot was immunostained using the highly-specific antibody 8B6, which recognizes both PS and DM proteins. The 50 kDa band is the expressed precursor PS and DM proteins and the 48 kDa band is the processed ("mature") protein. Densitometric analysis of mature PS and DM band densities, relative to the purified PS standard, supported estimates of 40.4 ng PS and 41.1 ng DM proteins in lanes 7 and 8 (1:1 ratio). Based upon total protein loadings in those lanes, PS and DM protein are estimated to be 1.4% and 1.3% of the total sample protein.

Table S1. PS- and DM-specific binding sites in promoters of potential target genes and expression patterns for those genes in PS- and DM-overexpressing Arabidopsis seedlings. Target genes for 5' binding sites are located upstream from the site, on the antisense strand. Target genes for 3' binding sites are located downstream from the site, on the sense strand. A 5'/3' binding site is located within divergent promoters of upstream and downstream target genes. Binding site data in divergent promoters are duplicated and are not counted in totals. Some genes have more than one PS- or DM-binding site.

Type of Binding Site	Location of Binding SIte	Number of Binding SItes	Potential Target Genes (Unique)	Specificity, Potential Target Gene Expression				Percent Target Genes Expressed	Percent Target Genes Specifically Expressed
				PS Seedlings	DM Seedlings	Both PS and DM Seedlings	Not DE		
PS	5'	2,915	2,692	74	24	9	2,586	4.1%	2.7%
	3'	2,701	2,527	66	29	11	2,421		
	5'/3'	520	988	26	5	2	922		
	**Totals:**	**5,616**	**5,219**	**140**	**53**	**20**	**5,007**		
DM	5'	4,029	3,658	107	39	10	3,502	3.8%	2.6%
	3'	3,915	3,515	83	28	7	3,397		
	5'/3'	783	1,472	36	10	3	1,374		
	**Totals:**	**7,944**	**7,173**	**190**	**67**	**17**	**6,899**		

Table S2. Differentially-expressed genes enriched specifically in an Arabidopsis DM-overexpression line are involved in abscisic acid (ABA) signaling or responses to ABA (BioProcess categories GO:0009737; GO:0009269).

AGI	Gene ID	Description	Expression (FC)		Notes
			PS	DM	
AT2G38310	PYL4	Abscisic acid receptor PYRABACTIN RESISTANCE-LIKE 4		1.5	Mediates ABA-dependent regulation of protein phosphatase 2Cs ABI1 and ABI2; stimulates ABA signaling
AT5G53160	PYL8	Abscisic acid receptor PYRABACTIN RESISTANCE-LIKE 8		1.5	Mediates ABA-dependent regulation of protein phosphatase 2Cs ABI1 and ABI2; stimulates ABA signaling
AT5G05410	DREB2A	Dehydration-responsive element-binding protein 2A		1.8	Transcription factor that specifically binds to DRE/CRT cis elements of water stress-inducible genes; drought stress tolerance
AT2G42280	AKS3	ABA-responsive kinase substrate 3; transcription factor bHLH130		1.5	
AT3G02140	AFP4	ABI FIVE BINDING PROTEIN 4		1.6	Negative regulator of ABA and salt stress responses
AT3G30775	POX1, ERD5	Proline dehydrogenase 1; EARLY RESPONSIVE TO DEHYDRATION 5; mitochondrial		2.5	Expression induced by osmotic stress; promoter contains an L-proline-inducible element
AT3G18490	ASPG1	ASPARTIC PROTEASE IN GUARD CELL 1		-1.6	Functions in drought avoidance through abscisic acid (ABA) signalling in guard cells
AT2G39800	P5CSA	Delta-1-pyrroline-5-carboxylate synthase A, P5CSA; P5C synthase 1		-1.5	Catalyzes the rate-limiting enzyme in the biosynthesis of proline; induced by abscisic acid, osmotic stress
AT3G11410	PP2CA	Protein phosphatase 2C 37; PP2CA		-1.5	mRNA up-regulated by drought and ABA
AT5G59220	SAG113	Protein phosphatase 2C 78; SAG113		-1.6	Highly ABA-induced PP2C gene 1; negative regulator of osmotic stress and ABA signaling

Table S3. PS-specific binding sites and enriched transcription factor motifs in promoters of genes that are differentially-expressed only in PS Arabidopsis seedlings (NOTE: This is a separate Excel file.)

Table S4. qRT-PCR validation of *WRKY42* and *PER50* expression in RNA-seq datasets.

		qRT-PCR		RNA-seq
		FC Expression	*p*-value	FC
PER50	col-0	1		
	PS	3.24 ± 0.47	0.02	1.6
WRKY42	col-0	1		
	PS	1.87 ± 0.17	0.02	1.9

Table S5. Chromatin proteins or transcription factors that interact with 4Myc-tagged Arabidopsis PS or DM proteins, or 13Myc-tagged yeast PS or DM proteins, in co-IP assays.

Protein ID	UniProt	Gene ID	Annotation
Arabidopsis			
CYP19-1	Q38900	AT2G16600	Peptidyl-prolyl cis-trans isomerase CYP19-1
NUCL1	Q9FVQ1	AT1G48920	Nucleolin 1
SWIB2	O82326	AT2G14880	Uncharacterized protein with SWIB/MDM2 domain
SCL30	Q9SNB8	AT3G46600	Scarecrow-like protein 30
Yeast			
FPR3	P38911	YML074C	FK506-binding nuclear protein
NSR1	P27476	YGR159C	Nuclear localization sequence-binding protein
MSC1	Q03104	YML128C	Meiotic sister chromatid recombination protein 1

Table S6. Expression profiles for peroxidase genes regulated in response to ectopic PS or DM expression [14] or suppression of *AtAPY1/2* in the R2-4A line (Lim et al. 2014) [29] of Arabidopsis seedlings.

Gene	Relative Expression (FC)			Gene Symbol	Subcellular Location	Description
	PS	DM	R2-4A			
AT5G64120	1.8			PER71	ECM	Peroxidase 71; lignification in roots; defense response to fungus; induced by hypo-osmolarity
AT5G19880	1.6			PER58	ECM, endomembrane system	Peroxidase 58; response to ethylene stimulus
AT4G26010	1.6			PER44	ECM, endomembrane system	Peroxidase 44; response to oxidative stress
AT5G64100	1.5			PER69	ECM	Peroxidase 69; response to oxidative stress
AT5G17820	1.5			PER57	ECM, plasma membrane	Peroxidase 57; response to oxidative stress, root
AT5G67400	1.5			PER73	ECM, endomembrane system	Peroxidase 73, root hair specific 19; response to oxidative stress
AT4G37520	1.5			PER50	ECM, cytoplasmic	Peroxidase 50; response to oxidative stress
AT1G30870	1.5			PER7	ECM, endomembrane system	Peroxidase 7; response to oxidative stress
AT5G06720	-1.7		6.0	PER53	ECM, endomembrane system	Peroxidase 53; diverse roles in the wound response, flower development, and syncytium formation
AT2G18150	-1.8		21.8	PER15	ECM	Peroxidase 15; response to oxidative stress

Table S7. PCR primers and oligos used in this study.

Primer Name	Primer Sequence (5'-->3')
**qRT-PCR Validation of RNA-seq Expression**	
WRKY42-F	TGTTCGCAAGCAAGTGCAAC
WRKY42-R	GACATGGTGGAGCCTGAGAG
PER50-F	CGTGACGTCGCGGGTG
PER50-R	ATCCTAATGTGTGTGCCCCG
**Construction and Screening of 4Myc-PS and -DM Overexpression Lines**	
pENTR-F	CACCATGGAGCTCCTTATTAAAC
pENTR-R	CAAGTCTTCCTCGGAGATTAGC
**EMSA Oligos**	
PHM6 test-Sense	ATAATGTTACGCAGTTCCGAGCTTCGCTGACACGTGGGAGGTGGGATATAATATTTGCACGGGTTC
PHM6 test-Antisense	GAACCCGTGCAAATATTATATCCCACCTCCCACGTGTCAGCGAAGCTCGGAACTGCGTAACATTAT
PHM6 mutant-Sense	ATAATGTTACGCAGTTCCGAGCTTCGCTGACACGTGGGAGGTGGGATATAATATTTGCACGGGTTC
PHM6 mutant-Antisense	GAACCCGTGCAAATATTATATCCCACCTCCCACGTGTCAGCGAAGCTCGGAACTGCGTAACATTAT
WRKY42 test-Sense	AGTTATTTTAACTAATAAACAGATAAAAAGAAATAAAAAGTACTAAATTGGTAAATTATCGTAGTATCAT
WRKY42 test-Antisense	ATGATACTACGATAATTTACCAATTTAGTACTTTTTATTTCTTTTTATCTGTTTATTAGTTAAAATAACT
WRKY42 mutant-Sense	AGTTATTTTAACTAATAAACAGATAAAAAGAAATAGCCTACACTAAATTGGTAAATTATCGTAGTATCAT
WRKY42 mutant-Antisense	ATGATACTACGATAATTTACCAATTTAGTGTAGGCTATTTCTTTTTATCTGTTTATTAGTTAAAATAACT
PER50 test-Sense	TTCACTTTTCAACTTATCCCTCTCAATCCCCACCACCACCACTCTCTATTCCCTCATATAGATAGATCAT
PER50 test-Antisense	ATGATCTATCTATATGAGGGAATAGAGAGTGGTGGTGGTGGGGATTGAGAGGGATAAGTTGAAAAGTGAA
PER50 mutant-Sense	TTCACTTTTCAACTTATCCCTCTCAATCCCCAGTCCTATGACTCTCTATTCCCTCATATAGATAGATCAT
PER50 mutant-Antisense	ATGATCTATCTATATGAGGGAATAGAGAGTCATAGGACTGGGGATTGAGAGGGATAAGTTGAAAAGTGAA

Table S8. Quality control metrics for the Arabidopsis ChIP-seq assay.

ChIP-seq Library	Unique Reads	Relative Strand Correlation (≥ 0.8)	Normalized Strand Coeeficient (≥ 1.05)	Genome Coverage (≥ 30x)
Col-0 input	27,284,031	1.250	1.037	30.95
Col-0	24,545,208	1.213	1.025	29.35
PS input	32,759,144	1.300	1.043	36.89
PS	34,225,803	1.364	1.027	54.21
DM input	48,730,589	1.291	1.028	41.00
DM	34,432,616	1.317	1.025	40.57
